# Editorial: Growth, peaking, and aging of competitive athletes

**DOI:** 10.3389/fphys.2023.1165223

**Published:** 2023-02-22

**Authors:** Hirofumi Tanaka, Jean-François Toussaint

**Affiliations:** ^1^ Department of Kinesiology and Health Education, The University of Texas at Austin, Austin, TX, United States; ^2^ Institut de Recherche bioMédicale et d’Épidémiologie du Sport (IRMES), Institut National du Sport, de l’Expertise et de la Performance (INSEP), Paris, France

**Keywords:** developement, masters athlete, athletic performance, veteran athlete, youth sport, peak performance

Athletic performance undergoes a complex trajectory of changes from birth to adulthood (Bergeron et al., 2015). Starting from peak levels achieved during early adulthood, performance declines progressively with aging (Tanaka and Seals, 2003). Numerous publications have explored the changes in athletic performance with age (Akkari et al., 2015; Berthelot et al., 2019). The primary goal of this Research Topic of Growth, Peaking, and Aging of Competitive Athletes was to capture a variety of studies dealing with the physiological determinants of performance development in youth, peaking in early adulthood, and aging with later declines.

Our Research Topic showcases several intriguing studies, including an examination of the relative age effect. This effect refers to the advantage that youth athletes born earlier in the cohort have in terms of performance and talent recognition (Berthelot et al., 2019). A study by Difernand et al. found that the relative age effect was prevalent consistently in all track and field events and became more pronounced at higher competitive levels. Based on careful analysis of time lapses between athletes’ birthdays and competition dates, the authors proposed rebalancing methods that compensate for the biases and validated such approaches using the Wilcoxon statistical test. This is one of the ways that youth performances can be better appreciated and evaluated by reducing the impact of relative age effect.

The age of peak athletic performance varies widely across different sports and between sexes/genders, typically ranging from 20 to 30 years old. While the peak performance age in men has remained remarkably stable since the first Olympic games in 1896, it has increased in women over the past 20–30 years (Elmenshawy et al., 2015). Alpine skiing, in particular, is a relatively under-researched sport in the realm of winter Olympic sports. A study by De Larochelambert et al. analyzed competition data from the French Ski Federation and found that the peak performance age for this sport was 24.8 years, similar to other summer sports. By using a data-driven approach, the researchers were able to identify four different types of progression curves that are clustered according to the performance level and progression per age. The results of this study will help athletes and coaches to better detect and predict potential of Alpine skiers.

The physiological and psychological changes that occur during the menstrual cycle and hormonal contraception have long been recognized as having large impact on athletic performance (Meignie et al., 2021). However, research in this area is very limited when it comes to elite female athletes. Antero et al. demonstrated that elite rowers with natural cycles had better performance and wellbeing in the middle of their cycle. Elite rowers taking hormonal contraception had better performance when they were taking pills. These findings highlight the need for greater awareness of the impact of menstrual cycle and hormonal contraception on athletic performance in elite female athletes.

There has been an increasing number of case studies focusing on elite athletes in general and older elite athletes in particular (Billat et al., 2017). Because these athletes are exceptional, it is not plausible to gather enough “n” to generate original investigation studies. Van Hooren and Lepers studied a 71-year-old age-group marathon world record holder who completed the race in 2:54:19. His maximal oxygen consumption (VO_2_max) of 46.6 mL/kg/min was exceptional for his age, and his marathon pace corresponded to 88.5% of his VO_2_max. However, these values were slightly lower than the previous age-group marathon record holder reported in a previous case study (Robinson et al., 2019). How did he beat the previous record holder’s time in spite of the lower ratings on key physiological determinants? Two remarkable findings were his vastus lateralis having over 90% slow twitch fibers and the training distance averaging over 130 km (80 miles) a week for the past 2 years. These two elements were linked to a better running economy in this remarkable runner.

By taking a holistic approach and exploring a wide range of research methods, this research area has the potential to provide valuable insights into the changes in athletic performance and its physiological determinants over time, and to inform interventions to enhance athletic performance throughout life. There are a number of research areas that should be explored in the future. For example, in light of recent discussion and awareness on mental health among elite athletes of all ages, impact of stress, anxiety, and other mental health on athletic performance should be investigated. Considering the increasing emphasis on diversity, equity, and inclusion (DEI), what is the impact of socioeconomic status on athletic performance particularly in youth and masters athletes? Future research could explore the impact of access to training facilities, equipment, and coaching on potential for athletic success. The future of this research area is bright.
